# A practical method for preparation of pneumococcal and nontypeable *Haemophilus influenzae* inocula that preserves viability and immunostimulatory activity

**DOI:** 10.1186/1756-0500-6-522

**Published:** 2013-12-09

**Authors:** Lea-Ann S Kirkham, Karli J Corscadden, Selma P Wiertsema, Andrew J Currie, Peter C Richmond

**Affiliations:** 1 School of Paediatrics and Child Health, The University of Western Australia, 35 Stirling Highway, Perth, WA 6009, Australia; 2 Telethon Institute for Child Health Research, Centre for Child Health Research, 100 Roberts Road, Perth, WA 6008, Australia; 3 School of Veterinary and Life Sciences, Murdoch University, South Street, Perth, WA 6150, Australia

**Keywords:** Ethanol-killed, Heat-killed, Live bacteria, Cryopreservation, *S. pneumoniae*, NTHi, Immunostimulation, PBMCs

## Abstract

**Background:**

Convenience is a major reason for using killed preparations of bacteria to investigate host-pathogen interactions, however, host responses to such preparations can result in different outcomes when compared to live bacterial stimulation. We investigated whether cryopreservation of *Streptococcus pneumoniae* and nontypeable *Haemophilus influenzae* (NTHi) permitted investigation of host responses to infection without the complications of working with freshly prepared live bacteria on the day of experimental challenge.

**Findings:**

*S. pneumoniae* and NTHi retained >90% viability following cryopreservation in fetal calf serum for at least 8 weeks. Host responses to live, cryopreserved (1 week and 4 weeks), heat-killed or ethanol-killed *S. pneumoniae* and NTHi were assessed by measuring cytokine release from stimulated peripheral blood mononuclear cells (PBMCs). We found that cryopreserved bacteria, in contrast to heat-killed and ethanol-killed preparations, resulted in comparable levels of inflammatory cytokine release from PBMCs when compared with fresh live bacterial cultures.

**Conclusion:**

Cryopreservation of *S. pneumonia*e and NTHi does not alter the immunostimulatory properties of these species thereby enabling reproducible and biologically relevant analysis of host responses to infection. This method also facilitates the analysis of multiple strains on the same day and allows predetermination of culture purity and challenge dose.

## Background

Many research laboratories, by necessity, use heat-killed, ethanol-killed, UV-irradiated or paraformaldehyde-fixed bacterial preparations to investigate host-bacterial interactions. However, stimulation of host immune cells with such inactivated preparations can result in very different outcomes when compared with live bacterial stimulation [1-3]. We have shown that in comparison with live *Staphylococcus epidermidis*, heat-killed and ethanol-killed *S. epidermidis* preparations have a reduced capacity to activate key innate immune pathways, especially those associated with cytosolic/endosomal bacterial recognition [3]. In addition, Mogensen and colleagues have demonstrated that live but not heat-killed preparations of *Streptococcus pneumoniae* and *Neisseria meningitidis* stimulated the host inflammatory response through Toll-like receptor 9 [2].

Convenience is a major reason for using killed preparations of bacteria to investigate host-pathogen interactions. Working with live bacteria usually requires growth to mid-log phase on the day of the stimulation experiment to ensure consistent and reproducible host responses. The time required for mid-log growth on the day of experimentation varies for different bacteria and can take up to 8 hours, which restricts the number of strains that can be assessed on one day and the time available for experimental challenge. In addition, culture contamination is usually only apparent on the day after experimental challenge of the host cells/animal models by checking purity of the culture on an agar plate incubated overnight. An alternative to broth cultures is to harvest bacteria from an overnight agar plate and resuspend in media to the desired optical density, which roughly correlates with bacteria/ml [4]. However, this means that the majority of bacteria are either in stationary phase or indeed dead when used to assess the host response, and results can vary accordingly. We herein describe a simple cryopreservation method using fetal calf serum (FCS) to store mid-log phase *S. pneumoniae* and NTHi for at least 8 weeks without a significant reduction in viability. We have used a PBMC stimulation assay to demonstrate that preparations of *S. pneumoniae* and NTHi frozen for up to 4 weeks retain the immunostimulatory properties of freshly prepared live bacterial preparations, whereas heat-killed and ethanol-killed preparations do not.

## Findings

**Figure 1:**
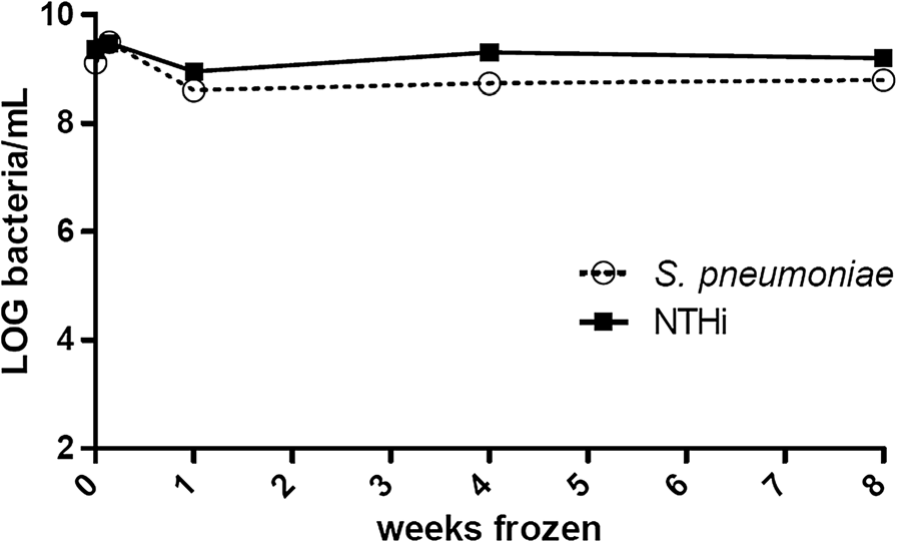
**Viability of *S. pneumoniae *and NTHi over 8 weeks of storage at -80°C.** The count on day 0 is a total bacteria count using a chamber (bacteria/ml), thereafter viable counts were conducted with the frozen bacteria to give LOG cfu/ml at 1 day, 1 week, 4 weeks and 8 weeks post-freezing.

There was a dip in viability when *S. pneumoniae* and NTHi were initially frozen, however, both species retained over 90% viability following 8 weeks cryopreservation (92.6 and 97.0% respectively when compared with 1 day of cryopreservation), Figure 1. Viability at 16 weeks cryopreservation was also measured and found to still be >90% for both species (data not shown).

**Figure 2:**
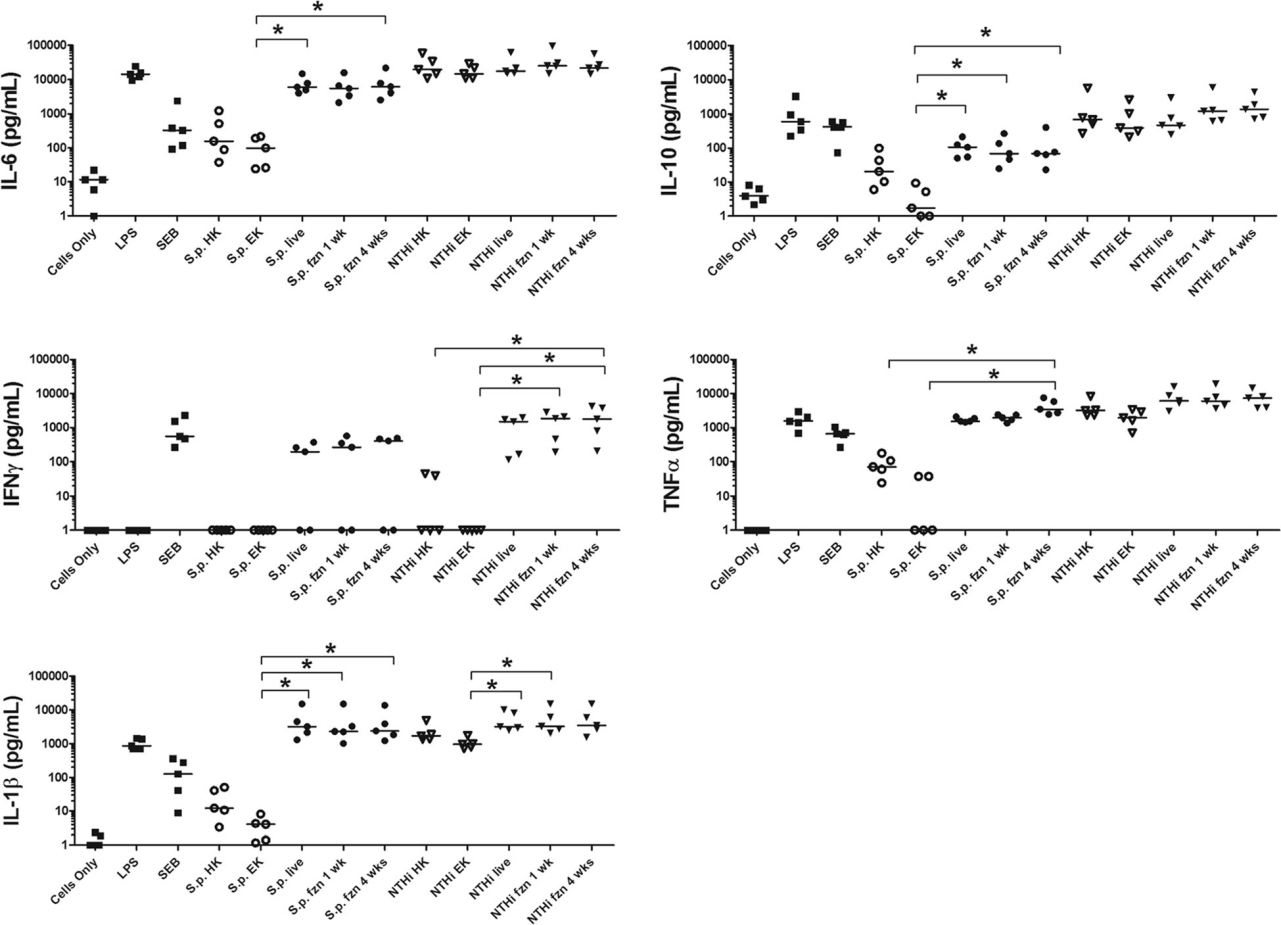
**Cytokine release (pg/ml) from adult PBMCs following stimulation with live, killed or cryopreserved bacteria.** Adult PBMCs were stimulated for 24 h with 1 × 10^7^ bacteria/ml of live, frozen 1 week (fzn 1wk), frozen 4 weeks (fzn 4wk), heat-killed (HK) or ethanol-killed (EK) *S. pneumoniae* and nontypeable *H. influenzae* (NTHi), lipopolysaccharide (LPS), or Staphylococcal enterotoxin B (SEB). The horizontal bars depict the median cytokine level for each treatment group (n = 5), * = P < 0.05 when compared with live or frozen bacteria.

With assurance that *S. pneumoniae* and NTHi remained viable following cryopreservation, we then challenged PBMCs from 5 adult donors with preparations of bacteria that were either frozen for 1 or 4 weeks and compared this with PBMCs challenged with heat-killed, ethanol-killed or live preparations prepared on the day of challenge. PBMC release of 5 inflammatory cytokines was measured as an indication of the immunostimulatory properties of the bacterial preparations. We found that there was no difference in the immunostimulatory properties of frozen *S. pneumoniae* and NTHi compared with live bacteria regardless of whether the bacteria were stored at -80°C for 1 or 4 weeks (Figure 2). In contrast, stimulation of PBMCs with ethanol-killed preparations resulted in production of significantly lower levels of IL-6, IL-10, TNFα and IL-1β for *S. pneumoniae,* and IFNγ and IL-1β for NTHi, when compared with live or frozen preparations (Figure 2, P < 0.05). Heat-killing retained slightly more immunostimulatory properties than ethanol-killing but there was still reduced immunostimulation in comparison with live or frozen bacteria. No IFNγ was released from PBMCs stimulated with either heat or ethanol-killed *S. pneumoniae* preparations, whereas an average of 200 pg/ml IFNγ was released upon stimulation with live or frozen *S. pneumoniae*. For NTHi, the IL-6, IL-10 and TNFα response was not dependent upon the bacterial treatment with high levels of cytokine production from live, frozen and killed preparations.

## Conclusions

We have described a simple and practical method that enables investigation of live host-pathogen interactions without the restrictions that are normally associated with working with live bacteria such as experimental time, contamination, intra-assay variation and scalability. Serum is a known microbial cryoprotectant [5] and although a similar storage method has been used with *S. pneumoniae* for challenge of mice [6] we have provided a detailed methodology and clearly demonstrated that cryopreservation of *S. pneumoniae* and NTHi with FCS preserves the immunostimulatory properties of these species. We have also confirmed that cryopreservation is superior to other methods for standardisation and storage of bacteria that involve inactivation.

Different methods of killing bacteria can alter the immunostimulatory profile of the pathogen either by exposing or destroying PAMPs [1-3]. This was evident in our study where heat and ethanol treatment of *S. pneumoniae* but not NTHi attenuated the IL-6 and IL-10 response from PBMCs. This is most likely to be due to the killing treatments destroying key pneumococcal virulence factors such as pneumolysin [1] but not lipooligosaccharide from NTHi, which is heat-stable. This highlights how using killed preparations of bacteria can result in an under or over-stated host immune response to the remaining immunostimulatory components and may result in misleading conclusions about host–pathogen interactions. In summary, we have developed a straightforward and convenient storage method for bacteria and demonstrated that cryopreserved bacteria remain viable for at least 8 weeks and maintain their stimulatory capacities for at least 4 weeks (later time points were not tested). This technique facilitates the analysis of multiple bacterial species on the same day, allows predetermination of culture purity and viability, and most importantly enables accurate investigation of the host response to live bacterial infection.

## Methods

### Bacterial strains, culture and cryopreservation

All reagents were from Sigma Aldrich, New South Wales, Australia unless otherwise stated. Glycerol stocks of *S. pneumoniae* D39 (ATCC#7466) and NTHi 289 [7] were streaked out onto blood agar or chocolate agar plates respectively for single colonies and incubated at 37°C. *S. pneumoniae* was incubated anaerobically using the BD GasPak™ EZ Anaerobic Pouch System [BD Diagnostics, Australia]. Following overnight incubation, three colonies were selected from each agar plate and used to inoculate culture media. *S. pneumoniae* was grown statically in brain heart infusion (BHI) broth at 37°C and NTHi was grown with shaking in BHI supplemented with 44 mL/L glycerol, 30 mg/L hemin and 10 mg/L nicotinamide adenine dinucleotide (NAD). Both strains were grown to mid-log phase (OD_600nm_ 0.55 – 0.65), counted at 100 × magnification using a Helber bacteria counting chamber [ProSciTech, Queensland, Australia] and then either subjected to the different treatments (heat, ethanol or freezing) or used fresh on the day of preparation. All cultures were streaked onto agar plates and incubated at 37°C overnight to check purity. For cultures that were to be frozen, heat-inactivated FCS [SAFC Biosciences, New South Wales, Australia] was added to the mid-log phase culture to give a final concentration of 20% FCS and 1 mL aliquots were stored in cryovials at -80°C. FCS was the only cryoprotectant used for storage of these bacterial stocks.

### Viable counts of frozen bacteria

Vials of frozen bacterial stocks were thawed at 37°C for 2 min in a water bath, 900 μL was transferred to a fresh tube and centrifuged in a benchtop centrifuge at maximum speed for 3 min. The supernatant was discarded and pellet resuspended in 900 μL of sterile phosphate buffered saline (PBS) pH 7.4 [Gibco, New South Wales, Australia]. Ten-fold dilutions of each bacteria ranging from 10^-1^ to 10^-6^ were prepared with PBS in a 96-well polystyrene round bottom plate. Agar plates were divided into 6 sectors, using 2 plates per strain, and three 20 μL aliquots of the dilutions were spotted onto each sector. Plates were allowed to dry then incubated overnight at 37°C. The following day, the number of colony forming units (cfu) per 20 μl spot were counted in the sector with approximately 20 – 80 cfu, averaged and multiplied by the dilution factor to give cfu/mL. Viable counts of cryopreserved bacteria were conducted at 1 day, 1 week, 4 weeks, 8 weeks and 16 weeks post-freezing.

### Heat-killing and ethanol-killing of bacteria

Mid-log phase cultures of *S. pneumoniae* and NTHi were centrifuged at 3200 g for 10 min, washed in PBS, and viable counts were conducted as described above. For heat-killing, the bacteria in PBS were incubated in a water bath at 60°C for 1 h. For ethanol-killing, the bacteria were resuspended in 70% ethanol and incubated at 4°C for 1 h with rotation. After killing, bacteria were washed in PBS, aliquoted and stored at 4°C. Chamber counts were conducted on the killed preparations to determine bacteria/mL and viable counts were conducted to confirm successful killing.

### PBMC collection, processing and stimulation with live, frozen, heat-killed and ethanol-killed preparations of bacteria

Whole blood was collected from five healthy adult donors by peripheral venepuncture and mixed 1:1 with heparinised (2%) RPMI 1640 media supplemented with 1% sodium pyruvate, 1% glutamax, 10 mM HEPES buffer [All Gibco]. The PBMCs were processed and stored as previously described [3]. On the day of stimulation, PBMCs were thawed at 37°C for 2 min in a water bath, added to 2% FCS RPMI 1640 and centrifuged at 500 g for 10 min at room temperature. The supernatant was discarded and cells resuspended in media and counted. The PBMCs were seeded in triplicate for each stimuli at 2.5 × 10^5^ cells per well in 96-well polypropylene round-bottom plates and incubated for 24 h at 37°C in a humidified 5% CO_2_ environment. PBMCs were stimulated with either live, heat-killed, ethanol-killed, frozen for 1 week or frozen for 4 week preparations of *S. pneumoniae* D39 and NTHi 289 at a multiplicity of infection of 10:1 bacteria to cells. Live and frozen bacteria viable counts were determined as described above and total bacterial chamber counts were conducted for the heat- and ethanol-killed preparations. Cells in control wells were stimulated with either PBS (cells only), 1 ng/mL lipopolysaccharide (LPS, from *E. coli* R515) [Alexis Biochemicals, Sapphire Biosciences, NSW, Australia] or 1 μg/mL staphylococcal enterotoxin B (SEB, from *S. aureus*). At 24 h post-stimulation, plates were centrifuged for 5 min at 200 g and supernatants were harvested, triplicate wells combined and then stored in aliquots at -80°C for subsequent measurement of inflammatory mediators.

### Measurement of inflammatory mediators

IL-6, IL-10, IFNγ and TNFα levels (pg/mL) were measured in cell culture supernatants using a previously described multiplex cytokine bead assay [8]. IL-1β levels in cell supernatants were measured using an ELISA kit [Bender MedSystems, California, USA] and following the manufacturer’s instructions, except wells were coated with an alkaline coating buffer (40 mM NaCo_3_; 70 mM NaHCO_3_) instead of PBS.

### Statistical analyses

PBMC cytokine release following different treatments was compared using a Kruskal-Wallis test with Dunn’s post-test analysis using GraphPad Prism [GraphPad Software Inc, California, USA], where P < 0.05 was considered statistically significant. For IL-1β one outlier was excluded from analysis due to an out of range maximum value.

## Abbreviations

Cfu: Colony forming units; EK: Ethanol-killed; HK: Heat-killed; IFN: Interferon; IL: Interleukin; NTHi: Nontypeable *Haemophilus influenzae*; LPS: Lipopolysaccharide; PAMPs: Pathogen-associated molecular patterns; PBMC: Peripheral blood mononuclear cells; SEB: Staphylococcal enterotoxin B; TNF: Tumour necrosis factor.

## Competing interests

The authors declare that they have no competing interests.

## Authors’ contributions

LK designed the study. LK and SW coordinated the study. KC conducted the viability checks, PBMC stimulations and cytokine measurements. SW, LK, AC and PR contributed to data interpretation and helped draft the manuscript. All authors read and approved the final manuscript.
